# A Pragmatic Benchmarking Study of an Evidence-Based Personalised Approach in 1938 Adolescents with High-Risk Idiopathic Scoliosis

**DOI:** 10.3390/jcm10215020

**Published:** 2021-10-28

**Authors:** Stefano Negrini, Sabrina Donzelli, Francesco Negrini, Chiara Arienti, Fabio Zaina, Koen Peers

**Affiliations:** 1 Department of Biomedical, Surgical and Dental Sciences, University “La Statale”, 20122 Milan, Italy; Stefano.negrini@unimi.it; 2 IRCCS Istituto Ortopedico Galeazzi, 20161 Milan, Italy; 3 ISICO (Italian Scientific Spine Institute), 20141 Milan, Italy; sabrina.donzelli@isico.it (S.D.); fabio.zaina@isico.it (F.Z.); 4 IRCCS Fondazione Don Gnocchi, 20148 Milan, Italy; carienti@dongnocchi.it; 5 Department of Physical Medicine and Rehabilitation, University Hospital Leuven, 3000 Leuven, Belgium; koen.peers@uzleuven.be; 6 Department of Development and Regeneration, University of Leuven, 3000 Leuven, Belgium

**Keywords:** adolescent idiopathic scoliosis, shared decision-making, personalised approach, bracing

## Abstract

Combining evidence-based medicine and shared decision making, current guidelines support an evidence-based personalised approach (EBPA) for idiopathic scoliosis in adolescents (AIS). EBPA is considered important for adolescents’ compliance, which is particularly difficult in AIS. Benchmarking to existing Randomised Controlled Trials (RCTs) as paradigms of single treatments, we aimed to check the effectiveness and burden of care of an EBPA in high-risk AIS. This study’s design features a retrospective observation of a prospective database including 25,361 spinal deformity patients < 18 years of age. Participants consisted of 1938 AIS, 11–45° Cobb, Risser stage 0–2, who were studied until the end of growth. EBPA included therapies classified for burdensomeness according to current guidelines. Using the same inclusion criteria of the RCTs on exercises, plastic, and elastic bracing, out of the 1938 included, we benchmarked 590, 687, and 884 participants, respectively. We checked clinically significant results and burden of care, calculating Relative Risk of success (RR) and Number Needed to Treat (NNT) for efficacy (EA) and intent-to-treat analyses. At the end of growth, 19% of EBPA participants progressed, while 33% improved. EBPA showed 2.0 (1.7–2.5) and 2.9 (1.7–4.9) RR of success versus Weinstein and Coillard’s studies control groups, respectively. Benchmarked to plastic or elastic bracing, EBPA had 1.4 (1.2–1.5) and 1.7 (1.2–2.5) RR of success, respectively. The EBPA treatment burden was greater than RCTs in 48% of patients, and reduced for 24% and 42% versus plastic and elastic bracing, respectively. EBPA showed to be from 40% to 70% more effective than benchmarked individual treatments, with low NNT. The burden of treatment was frequently reduced, but it had to be increased even more frequently.

## 1. Introduction

The practice of evidence-based medicine (EBM) combines evidence with physicians’ expertise and patients’ values [1]. As it comes mainly from the results of Randomised Controlled Trials (RCTs) focused on single treatments, a contradiction has been suggested between RCTs and personalised medicine/shared decision making [2,3], which has been shown to be important [4,5]. This contradiction also exists in clinical practice, where some practitioners prefer to follow strict protocols, and others propose highly personalised approaches. Extensive observational prospective studies can verify personalised approaches from a realistic everyday perspective [6] and verify the generalisability of RCTs [7]. In the case of conservative treatment of adolescent idiopathic scoliosis (AIS), RCTs [8,9,10] showed the efficacy of single treatments. Nevertheless, current clinical guidelines support an evidence-based approach, which clinicians can personalise within a range of different possible treatments for each clinical condition [11]: in this paper, we call this approach an evidence-based personalised approach (EBPA). EBPA is particularly advocated for adolescents because they are neither children doing what parents impose nor adults performing conscious choices. Still, they need to share decisions to adhere to treatments [11].

Scoliosis is a three-dimensional deformity of the spine and trunk with a prevalence of 2–3% in the general population [11]. The most common idiopathic type is classified according to the age at discovery, being most frequent in adolescence (AIS). AIS can have an aesthetic impact and cause in adulthood progressive deformities and back pain. Gold standard measures are Cobb degrees on a posteroanterior full-spine radiograph [11] where scoliosis is diagnosed as >10°, and health problems in adulthood are common at >50° and unusual at <30° [12,13,14]. A substantial percentage of adolescents rapidly progress during growth, with high risk between age ten and Risser bone maturity stage 2 [14,15].

The Bracing AIS Trial (BrAIST) RCT [8] confirmed the efficacy of plastic thoraco-lumbo-sacral orthosis (TLSO) for AIS of 20–40° consistently with a previous benchmarking controlled trial [16]. Results from cohort studies showed a various range of results from no efficacy to very high efficacy [17,18]. Population selection, research methodologies, patients’ compliance, brace type, and construction, as well as expertise and management skills [11,19], can explain these differences. Minor side effects have been reported [8,20], with psychological impacts only occurring for braces extending to the cervical region [8,11,21]. Two RCTs have shown the efficacy of physiotherapeutic scoliosis-specific exercises (PSSE) [10] and elastic bracing (SpineCor) [9] in curves of 15–25° and 15–30°, respectively. The Cochrane Systematic Review on PSSE [22] is under revision to include other short-term RCTs that confirm the efficacy of PSSE, together with a pragmatic perspective [23] and one other design [24] study. Side effects have not been reported. There is no evidence for other conservative treatments [11].

Bracing and PSSE are demanding treatments proposed for asymptomatic adolescents to avoid curve progression to the reported risk thresholds [14]. These treatments last years until the end of growth. Consequently, compliance is one of the most significant issues [8,11,25]. Current clinical guidelines propose by consensus a range of possible treatments for each clinical condition, which experts must individually tailor through shared decision making. They also propose what we call here EBPA to achieve the best results to reduce the treatment burden and increase compliance through patients’ adherence: this is achieved through a step-by-step path aimed to provide the most effective treatment with the lowest impact. Finally, they stress over- and under-treatment as well-known mistakes, since they cause unnecessary burden on patients or curve progression, respectively [11].

Nevertheless, we are not aware of any study on EBPA for AIS. We aimed to verify its results in a prospectively collected broad cohort of high-risk (Risser 0–2) AIS patients. We also wanted to compare two possible models of treatment diffused in the clinical world: EBPA versus per-protocol. This was done comparing existing RCTs (as a paradigm of per-protocol treatments) to subgroups of our EBPA cohort benchmarked (matched) for inclusion criteria. We expect these results to contribute to the debate about personalised decision making versus per-protocol approaches, particularly in adolescence when a personalised approach could be more appropriate than in adulthood. We also expect to verify the current evidence on AIS treatments generalising RCTs to everyday practice and determine the feasibility and importance of an EBPA.

## 2. Materials and Methods

### 2.1. Study Design and Participants

We designed a retrospective observational study nested in a prospective clinical database including all prospectively collected data of patients of a tertiary referral institute. The institute is specialised in the rehabilitation (conservative treatment) of spinal disorders at all ages, with specific attention to idiopathic scoliosis during growth. The prospective clinical data collection started in March 2003. At the time of data collection (31 December 2017), we included 29,859 individuals with spinal disorders, with 25,361 having had the first consultation before age 18.

**Figure 1:**
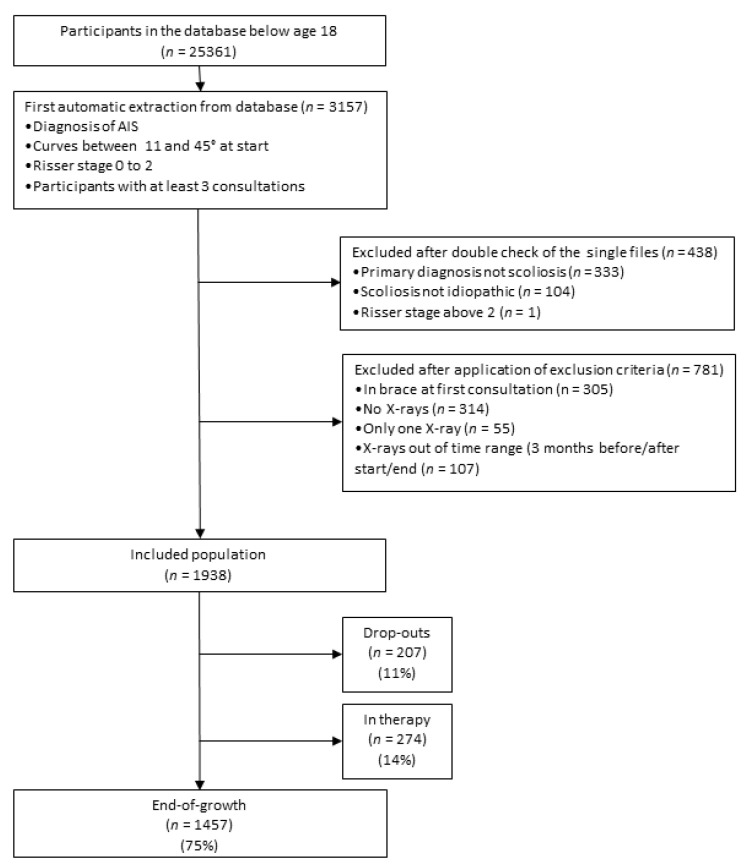
Selection of patients. Flow-chart of selection of participants from the clinical perspective. database. AIS, adolescent idiopathic scoliosis.

We defined the following inclusion criteria: AIS diagnosis [11], curves 11–45° at the start, and a Risser stage between 0 and 2. Our institute receives many patients for a second opinion, so we only included those in charge, which we defined as adolescents who came at least three times to our facilities. The exclusion criteria were wearing a brace at first consultation and absence of X-rays in the three months before or after the start or end of treatment and observation. We found 1938 participants that fit the inclusion criteria (Figure 1). To compare the EBPA proposed in this study to standard treatments provided by RCTs, we selected three subgroups of participants paired to the existing end-of-growth RCTs using their inclusion criteria (Table 1). The Plastic Bracing (PB), Elastic Bracing (EB), and PSSE subgroups were compared to the BrAIST [8], SpineCor [9], and Monticone [10] papers, respectively. The 3 subgroups included 3 subsamples of the entire observed cohort of 1938 adolescents of 687, 884, and 590 participants, respectively,

All parents provided written informed consent. The local Ethical Committee (Comitato Etico Milano Area B, Via F. Sforza 28, Milan, Italy-parere 801_2015bis, 15 December 2015) approved the study protocol, which is available at clinicaltrials.gov. 

### 2.2. Procedures

Participants underwent a medical evaluation every 4–6 months, according to their growth rate. We prescribed a radiographic exam every two consultations, measuring Cobb degrees and recording the Risser stage.

Proposed treatments included observation, PSSE, and bracing. We proposed treatments according to the current situation and progression risk as determined by physician expertise by combining risk factors including Cobb degrees, growth, history, angle of trunk rotation (ATR), sagittal plane measures (radiographic measures of kyphosis and lordosis, pelvic parameters, and plumbline distances), aesthetics evaluated through the Trunk Aesthetic Clinical Evaluation (TRACE) scale, and others (e.g., family history) [11]. We proposed observation in low-degree, low-progression risk AIS to verify the effect of growth. PSSE followed mainly the SEAS (Scientific Exercises Approach to Scoliosis) approach [26], even if some participants autonomously chose other techniques; exercises were used in patients with a low degree and low to medium progression risk AIS to avoid bracing. An elastic brace (SpineCor 20 h/day) was used for 20–30° AIS not considered at high risk of progression. Plastic braces and included the rigid Sibilla, Lapadula [27], and PASB (Progressive-Action Short Brace) [28], and the very rigid [21] Sforzesco [27] braces. Plastic brace prescriptions ranged between 18 and 24 h/day according to progression risk. PSSE were always prescribed in combination with any brace prescription.

According to the SOSORT Guidelines [11], we defined the intensity of available treatments (Table 2) and a range of acceptable proposals for each clinical condition. These ranges fell within the range of possibilities proposed by the guidelines, so keeping an EBM approach. Each physician of our institute contributed to the definition of these protocols, which were gradually improved (as typical for EBM) with time, also with the introduction of new treatments (such as SpineCor since 2010). In front of single patients corresponding to each pre-defined clinical condition, physicians chose the treatment options remaining within the acceptable pre-defined range. The electronic patient record allowed checking the coherence between the protocols and the therapeutic proposals made by physicians, allowing to gradually improve the system. More information about the EBPA approach used in this study is reported in Appendix A.

To achieve an informed, shared decision, participants systematically received information from the treating physician about their clinical and aesthetic condition, progression risk, the importance of the 30°/50° thresholds [14], and how they could influence their health and possible results. We discussed alternatives to either reduce the burden of treatment or increase the probability of success. We finally proposed the prescription according to a risk/benefit ratio agreed upon with the patient and family. Hence, we can precisely describe the treatment provided only post hoc, since clinical decisions were always personalised. Appendix A provides examples of clinical decisions and the pathway followed by clinicians with patients to achieve the final individualised prescription. EBPA differs from a per-protocol approach in the quantity of information provided, full range of treatment alternatives proposed as effective, and the greater interaction with patient and family, with a final decision taken together on these bases.

After consultation, a trained scoliosis expert provided a cognitive–behavioural intervention (20–45 min) to answer questions and give information. An email question-and-answer service was also provided. In cases of bracing, in case of stability or improvement, a reward strategy was adopted with a gradual decrease every six months of 2 h/day. We kept an 18 h/day minimum dosage until Risser stage 3. Participants completed the study at bone maturity, defined as Risser stage 4, or at complete brace gradual weaning, if achieved after bone maturity.

### 2.3. Data Analysis

We analysed all data by sex, but it was not by race due to the uniformity of the Italian population. We defined the treatment intensity using an ordinal scale adapted from the current guidelines. We listed therapies in seven classes from the least (observation) to the most effective and burdening for participants (very rigid brace, 22–24 h/day) (Table 2). We used this scale to study the final results. We checked the scale application in our sample dividing all patients per treatment applied and verifying the Cobb degrees differences among groups.

Outcomes included the number of patients that achieved the primary outcomes of <30° and <50° [14]. Secondary outcomes included improvement or progression of >5° [14]. We also included achieving the current guidelines’ [11] primary (optimal) and secondary (minimum desired achievement) treatment aims. Finally, we faced the concepts of under- and over-treatment. They are classically described as mistakes in AIS management since they mismatch between finally achieved results and applied treatments. These concepts start from the premise that increasing treatment intensity (as defined above) means higher efficacy and more patient demands. Consequently, under-treatment describes a therapy not effective enough to achieve desired targets, and over-treatment describes the opposite: therapies demand could have been lowered, since the results achieved were above the needs of the patients. We considered under- and over-treatment compared to the most relevant target, achieving adulthood with a curve below 30° [12,13,14]. We defined under-treatment as (1) when patients started treatment below 30° if the deformity progressed above 30°, and (2) when patients started treatment above 30° if they progressed at all. There is not a generally accepted definition of over-treatment (too demanding therapies), which is a concept stated in the guidelines [11] but not operationalised. For this paper, we needed such an operationalisation. Consequently, we defined over-treatment as any unnecessary improvement identified through a specific formula based on the 5° Cobb radiographic measurement error (Table 3). Improvements could be considered unnecessary when not changing the future of patients according to the known threshold of 30° for future problems in adulthood [14].

We compared the PB, EB, and PSSE subgroups with the corresponding paired RCTs [8,9,10] for benchmarking purposes. We compared the baseline data using t-tests calculated using averages, standard deviations, and the number of patients. We used chi-square tests for percentages of different categories and final results. The outcomes for the paired RCTs were (1) ending at >50° for PB [8], (2) progression >5° for EB [9], and (3) progression >3 ° for PSSE [10]. We performed an efficacy analysis, considering all patients who reached the end of treatment, and an intent-to-treat analysis (ITT). In the ITT, we hypothesised a worst-case scenario, with the patients who dropped out interpreted as failures. For EBPA and the treated group of the compared RCTs, we computed Relative Risk (RR) of success, Number Needed to Treat (NNT), and 95% Confidence Interval (95CI). These parameters could be calculated because our sample and those of the RCTs had been collected prospectively. Moreover, the comparison groups of the RCTs could offer natural history data to be compared to our subgroups, providing a successful pairing with similar baseline parameters in our subgroups and RCTs participants.

We collected data through software developed by our institute, managed with Excel, and analysed statistically with STATA 13 Texas 77845 USA.

## 3. Results

**Figure 2:**
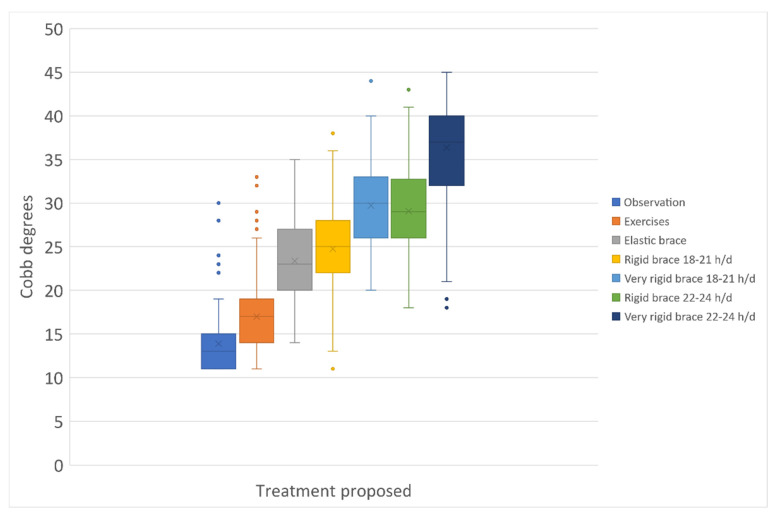
The intensity of treatments applied in the whole sample per Cobb degrees at the first consultation. All treatment groups were statistically different one from the other, with the only exception of “Very rigid bracing 18–21 h/day (h/d)” versus “Rigid bracing 22–24 h/d”, where other determinants beyond Cobb degrees could play a role. Data of each single subgroup compared to RCTs are reported in Table 4.

Out of 1938 participants, 274 (14%) were still in treatment and excluded, and 207 (11%) dropped out (Figure 1). We could not determine the reasons for dropping out due to the observational design of the study. The EBPA applied in the full cohort is described in Figure 2: each treatment group is statistically different from the others, with the exclusion of the comparison “Very rigid bracing (Sforzesco) 18–21 h per day” versus “Rigid bracing 22–24 h/day”. The EBPA applied in every single subgroup benchmarked to an RCT is reported in Table 4. We could not benchmark our PSSE subgroup, since it included less mature (Risser stage) and 10 cm smaller participants than the paired RCT. For EB and PB subgroups, we found a few statistically but not clinically significant differences with the intervention and observation arms of corresponding RCTs at the baseline (Table 4).

Table 5 reports the individual choices made at the first patient–physician encounter (EBPA) throughout the 18 years of observation according to each clinical condition (determined according to the degree of scoliosis and Risser sign—see Appendix A). There is a percentage of patients who had a higher or lower treatment intensity. The same information about the subgroups and their benchmarked studies are reported in Table 4.

Due to the inclusion criteria, we had at baseline 69% of patients < 30°. At the end of treatment, this percentage increased to 78%, with 2% progressing above 50°. Improvement occurred in 33% and progression occurred in 19%. We reached the primary (optimal) guideline aims in 68% and the secondary (minimal) in 98%. Under- and over-treatment occurred in 13% and 10%, respectively.

**Figure 3:**
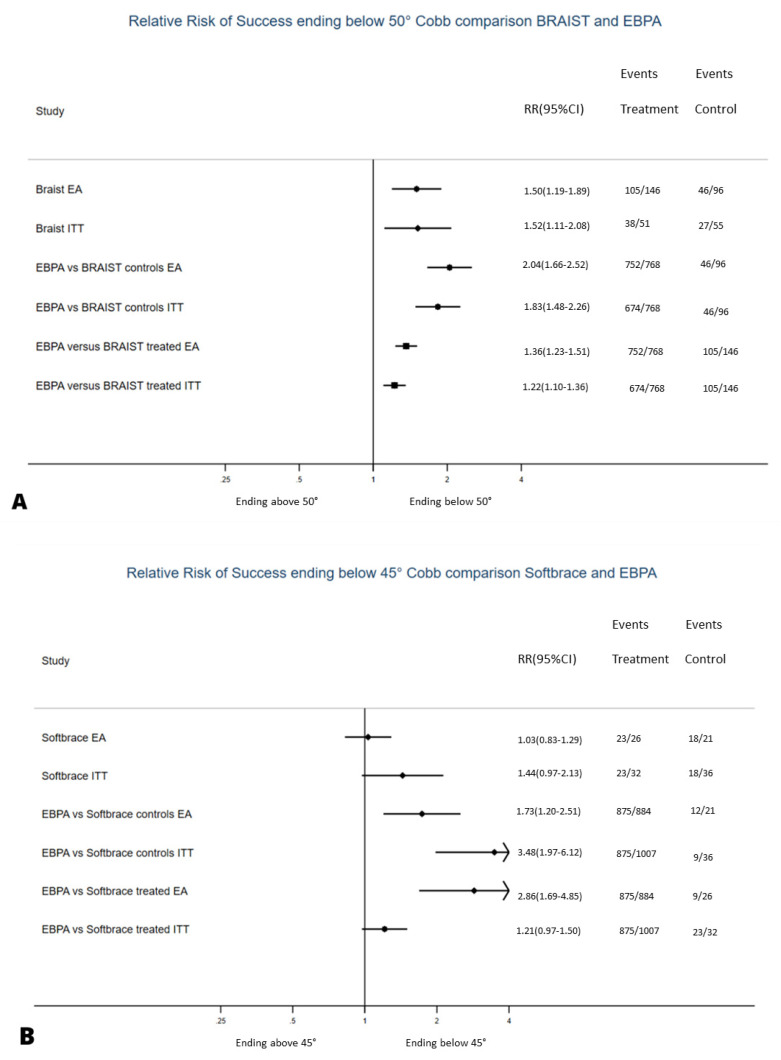
Relative Risk of success of the evidence-based personalised approach (EPBA) to paired RCTs [8,9]. Results in terms of Efficacy Analysis (EA) and Intention-to-Treat (ITT) are compared to the observational arms of each of the two studies. We used the Relative Risk (RR) of success since all data in RCTs and EBPA were collected prospectively. A higher RR shows the probability for a patient to achieve better results with one treatment vs. the other. The vertical line corresponds to the natural history data collected in every single RCT for the first four lines (for the original RCT, the first two lines, for the EBPA subgroups, the second two lines), while that in the last four corresponds to the comparison group coming from the RCTs (lines 3–4 to controls/natural history, lines 5–6 to the RCT treated group). The RCT on physiotherapeutic scoliosis-specific exercises (PSSE) [10] has not been compared since baseline populations were statistically significantly different. The same was true for the subgroups when we exclusively applied the treatment proposed in the RCTs.

To check the efficacy of EBPA, we benchmarked our subgroups to RCTs controls (Figure 3). For the efficacy analysis, EBPA had 2.0 (1.7–2.5) and 2.9 (1.7–4.9) RR of success, with 2 (1.7–2.5) and 2.4 (1.6–4.8) NNT for BrAIST and SpineCor studies, respectively (Table 6). Failure rates of PB and EB were 2% and 19%, respectively, versus 52% and 75% in the observed arms of the BrAIST and SpineCor studies, respectively.

**Figure 4:**
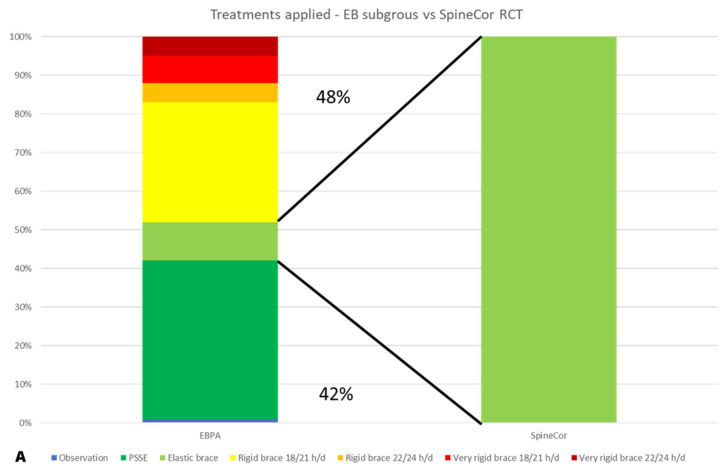

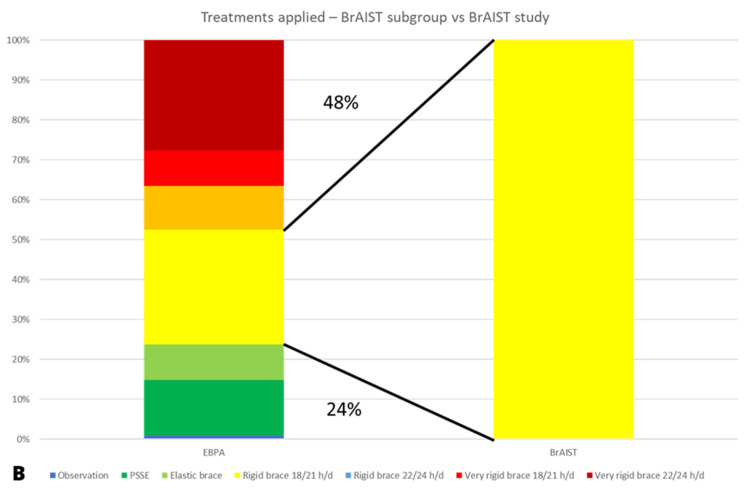
Comparison of the intensity of treatment for the evidence-based personalised approach (EPBA) versus paired RCTs [8,9]: (**A**) Thoraco-Lumbo-Sacral Orthosis (TLSO) [8] and (**B**) elastic braces [9]. The improvement in results of EBPA has been obtained using greater treatment intensity than paired RCTs in 48% of cases, but also a reduced treatment intensity in 24% of the BrAIST subgroup and 42% of the EB subgroup.

To compare EBPA to single treatments, we benchmarked our subgroups to the RCTs treated groups (Figure 2). For the efficacy analysis, EBPA had 1.4 (1.2–1.5) and 1.7 (1.2–2.5) RR of success, with 3.8 (2.9–5.3) and 1.6 (1.2–2.2) NNT for plastic and elastic bracing, respectively. The above reported 2% and 19% failure rates of PB and EB compared to 28% and 34% in the treated arms of the BrAIST and SpineCor studies, respectively. EBPA treatment burden was more significant than RCTs in 48% of patients and reduced in 24% and 42% versus plastic and elastic bracing, respectively (Figure 4).

## 4. Discussion

This pragmatic observational study of a large prospective cohort benchmarked to published RCTs shows a higher efficacy of EBPA than standardised protocols. The probability of success of patients treated in EBPA is between 1.5 and 3.5 times that of natural history and between 1.2 and 2.9 when compared to per-protocol treated groups (RR in Table 6). This higher efficacy than what was reported in RCTs corresponds to a reduced burden of treatment for a high percentage of patients but also more demands on another important percentage of patients. The dropout, over-, and under-treatment rates are significant: 10%, 10%, and 13%, respectively.

The results of this study could be due to the real-world pragmatic approach of the EBPA we used vs. the artificial process of a research environment of a per-protocol RCT. It could very well be true that a real-world clinic that uses a strict protocol for all patients shows similar good results just because patients are motivated and not subject to an artificial randomisation and study process. Nevertheless, the study could also support the idea that a personalised approach based on shared decision making is superior to a standardised protocol. The latter is consistent with some papers based on different methodologies and other fields of medicine [5]; it is also coherent with the strong support given nowadays to patient-centred care. This is probably significant in AIS and adolescence [11] and could be specific to the field, but that is not necessarily the case. Shared decision making could improve compliance [2,3,4,5] and consequently final results, which could be particularly important in clinical areas where compliance is a problem, as in AIS [11,19,25,29]. We disagree with the contradiction suggested between EBM and shared decision making [2,3]. RCTs are one of the means to achieve EBM [1], but they do not coincide with it. The complete per-protocol application of RCT results is quite frequent among clinicians but not supported by EBM, and in the field of AIS, it can be one of the factors leading to the disparity of results reported in the literature [11,17].

As in previous studies [8,11,21], bracing has shown to be highly effective; personalisation increases its efficacy while reducing invasiveness whenever possible. Other elements can contribute to EBPA to explain the current results. Measured compliance was comparable to some studies [29] but much higher than others [8,30], which could explain our results and could be due to EBPA or the cognitive–behavioural approach [25]. The braces that we used are the most symmetric reported in the literature [18] with the precise aim of reducing visibility and increasing compliance [27]. Moreover, other brace-related technical and biomechanical factors could explain our results. Finally, we must consider that our braced patients always practised PSSE, too. This could influence final results as well as compliance [11].

This study confirms the possibility of stabilising AIS with PSSE [22,24] according to a pragmatic trial [23], but not a previous RCT [10] that reported improvements. This RCT population was different from all the other RCTs and our study, and the possible reduced progression risk due to higher bone maturity precluded any benchmarking.

The issue of under- and over-treatment is discussed in the AIS community [11], but we are not aware of any numerical definition. We proposed a specific approach internal to our EBPA population based on the most desired target of conservative treatment: an outcome below the 30° threshold [14] or, if not possible due to the starting deformity, approaching it as much as possible. Of note, we could also compare EBPA to per-protocol standard RCTs approach using the same concept. In this case, the higher percentage of bad results in RCTs could be considered under-treatment (i.e., not enough efforts are required for patients to achieve better results). Conversely, good results obtained with less invasive procedures with EBPA could be considered over-treatment by the RCTs (i.e., treatment invasive than what required by the clinical situation—as shown by EBPA being a less demanding treatment). Using this approach, under-treatment and over-treatment account for 48% and 25% for the standards TLSO 18 h/day in 20–40° AIS [8], and 48% and 42% for SpineCor in 15–30° AIS [9], respectively.

We had a 10% dropout rate, which may be due to the demands and length of treatment. On average, treatment lasted 3.7 ± 2.1 years, while dropouts stopped after 1.8 ± 0.8 years. Future studies should verify if these patients stopped treatment and their results at the end of growth. Nevertheless, at dropout, their results were not different from the other patients, and consequently, considering them total failures as we did in the ITT analysis is questionable.

The strengths of our study include the representation of everyday clinical reality, the possibility of checking many different factors and research hypotheses, a focus on the highest-risk population, and the large numbers achieved through a specific database. While this study reports real-world results, we also have to consider that the EBPA we used was developed in a tertiary referral institute. AIS is usually treated in tertiary referral institutes, particularly when it becomes important, but this is not always the case. Consequently, we cannot consider these results generalisable to everyday clinical life but only to tertiary referral institutes where high specific competencies are retrievable.

The limitations of our study include the non-randomised design that increases the rate of confounders. Nevertheless, RCTs for AIS are becoming difficult due to very high costs, large effect sizes that lead BrAIST to stop recruitment for ethical reasons [8], high failure rates, and recruitment difficulties [21]. Since the quality of evidence on AIS treatment is between low and very low [21,22], this study contributes to strengthening current evidence. This paper includes all patients who came to our institute according to specifically defined criteria, being representative of daily practice in a wide group of patients. Conversely, the subgroups compared to RCTs may not completely represent the everyday clinical reality, since they could share one possible problem of data from RCTs, which is the limited inclusion criteria leading to selection bias. However, the subgroups do demonstrate the success percentage in treatment per-protocol in an RCT compared to EBPA. The specialisation of our institute could reduce generalisability, but expertise is a prerequisite for EBPA. It is also theoretically possible that the rigor of data collection in clinical everyday life was reduced when compared to a research setting such as in RCTs. There are two main reasons why this should not happen: the electronic medical records of the institute provide a quality check of all individual patients and treating physicians need all data reported in this paper for their clinical follow-up. The only possibility would be not to report on the existence of a radiograph, but this does not happen. Future studies should verify the current definition of over- and under-treatment.

## 5. Conclusions

EBPA showed to be from 40% to 70% more effective than benchmarked individual treatments, with low NNT. The burden of treatment was frequently reduced, but it had to be increased even more frequently. These results contribute to the clinical and research debate about personalised medicine based on shared decision making versus standard protocols, particularly in adolescence. They impact practitioners because they confirm data on the efficacy of bracing and PSSE and stress their importance as components of a personalised approach. Guideline developers should continue to keep the concept of personalisation also in future recommendations [11]. Health policy managers should value an expert approach to scoliosis by tertiary referral practitioners. Finally, reducing patients requiring more invasive and costly procedures is highly relevant, particularly in low to middle-income countries where access to some treatments can be challenging.

## Figures and Tables

**Table 1:** Inclusion criteria of the existing end-of-growth Randomised Controlled Trials (RCTs) used for benchmarking in the current study.

Subgroup	Plastic Brace TLSO	Elastic Brace (SpineCor)	Physiotherapeutic Scoliosis-Specific Exercises
Acronym	BrAIST	EB	PSSE
Comparison RCT	Weinstein et al. [8]	Coillard et al. [9]	Monticone et al. [10]
Age (years)	10–15	8–15	10 or older
Risser (grade)	0–2	0–2	0–1
Menarche	pre-menarchal or 1-year post-menarchal	-	-
Cobb angle	20–40°	15–30°	11–25°
Curve	apex at, or caudal to T7	-	-
Previous treatments	not for AIS	-	-

Results of the patients treated with an evidence-based personalised approach (EBPA) in this study have been compared to the standard treatments received in the compared RCTs. BrAIST: Bracing Adolescent Idiopathic Scoliosis Trial; TLSO: Thoraco-Lumbo-Sacral Orthosis.

**Table 2:** The intensity of treatment.

Intensity of Treatment	Treatment
0	Observation
1	Physiotherapeutic Scoliosis-Specific Exercises
2	Elastic brace (SpineCor)
3	Rigid plastic brace (Sibilla, Lapadula, PASB) brace 21 h/day or less
4	Rigid plastic brace (Sibilla, Lapadula, PASB) brace 22–24 h/day
5	Very rigid plastic brace (Sforzesco) brace 18–12 h/day
6	Very rigid plastic brace (Sforzesco) brace 22–24 h/day

In this table, treatments provided in the study have been ordered by intensity from the less to the most demanding. This corresponds also to the order from the least to the most effective. This order has been defined by expert consensus by the current SOSORT guidelines [11] and accepted by the SRS-SOSORT Consensus for research studies [14]. The ordinal scale used here has been adapted as follows: (1) night-time rigid bracing and scoliosis intensive rehabilitation were not present in our cohort and have been excluded; (2) according to the last Cochrane Review on bracing [21] we have differentiated between rigid and very-rigid braces; (3) half-time and part-time rigid bracing have been combined (categories 3 and 5) as well as full and total time (categories 4 and 6); (4) all observation categories with different time intervals between check-ups have been collapsed into the 0 category. PASB, Progressive-Action Short Brace. Note: the Sforzesco brace was developed between 2004 and 2005. Till then, the Lyon brace was the very rigid brace used.

**Table 3:** Aims of treatments, over-, and under-treatment according to the different clinical situations.

Clinical Situation at the Start		Aims of Treatment According to Current Guidelines [11]		Thresholds of Over- and Under-Treatment	
Degree of scoliosis	°Cobb	Primary aim	Minimal aim	Over-treatment	Under-treatment
Low	11–20	End of growth < 20°	End of growth < 45°	Improvement > 5°	End of growth > 30°
Moderate	21–25	End of growth < 30°		Improvement > $5{}^{{}^{\circ}}+\left(\frac{{}^{{}^{\circ}}\mathrm{Cobb}\;\mathrm{start}\;-20{}^{{}^{\circ}}}{2}\right)$	
	26–30				Progression > 5° #
	31–40				
Severe	41–45	End of growth < 45°	End of growth < 60° *		

Current guidelines [11] define the primary aim as the optimal desired achievement, while the minimal aim corresponds to the minimum desired achievement, in cases where it is impossible to obtain the primary aim. Keeping in mind the threshold of 30° [12,13,14], under-treatment has been defined when the deformity progressed above this significant threshold or, if already above, if it progressed at all. Conversely, over-treatment has been defined when there have been successful (1) improvements, for starting points below 21°; (2) important improvements according to a specific formula, reaching scoliosis below 30°, for starting points above 20°. * For severe curves, the guidelines propose postponing surgery [11]. We arbitrarily decided that a 60° curve requires immediate surgery, while below it is still possible to achieve results with conservative treatment [21] and set this threshold. # Only if therapy intensity was below 6.

**Table 4:** Comparison of baseline characteristics of subgroups BrAIST and EB in our study with paired RCTs [8,9]. Values are reported with ± Standard Deviation or 95% Confidence Intervals in parenthesis.

		BrAIST Subgroup vs. BrAIST RCT					EB Subgroup vs. Coillard RCT					PSSE Subgroup vs. Monticone RCT				
		BrAIST Subgroup of the Current Study	Comparison with BrAIST RCT Groups				Coillard Subgroup of the Current Study	Comparison with Coillard RCT Groups				PSSE Subgroup of the Current study	Comparison with Monticone RCT Groups			
			Observed	*p*	Treated	*p*		Observed	*p*	Treated	*p*		Observed	*p*	Treated	*p*
Number		687	96		146		884	36		21		590	55		55	
Age		12.7 ± 1.3	12.7 ± 1.2	NS	12.7 ± 1.0	NS	12.11 ± 1.5	12.2 ± 2	NS	12.2 ± 2	NS	12.6 ± 1.4	12.5 ± 1.1	NS	12.4 ± 1.1	NS
Female sex		83% (81–86)	90% (84–96)	0.13	92% (88–96)	0.01	82% (80–85)	86% (74–97)	0.68	85% (69–100)	0.88	82% (79–85)	71% (59–83)	0.07	75% (63–86)	0.23
Race	White	100%	76% (67–85)	<0.0001	79% (72–86)	<0.0001	100%	NA		NA		(100%)	NA		NA	
	Black	0	11% (11–11)		8% (8–8)		0	NA		NA		0	NA		NA	
	Other	0	9% (9–9)		5% (5–5)		0	NA		NA		0	NA		NA	
	Unknown	0	3% (3–3)		8% (8–8)		0	NA		NA		0	NA		NA	
Height		157.3 ± 9.1	153.6 ± 10.6	0.0002	156.5 ± 9.1	NS	158.7 ± 9.5					156.2 ± 9.1	146.3 ± 7.5	<0.0001	147.0 ± 5.7	<0.0001
Cobb angle of the largest curve		28.4 ± 5.8	30.3 ± 6.5	0.003	30.5 ± 5.8	<0.0001	22.3 ± 4.5	20.0 ± 4.1	0.002	22.0 ± 4.9	0.3	18.1 ± 4.1	19.3 ± 3.9	0.03	19.2 ± 2.5	0.05
Risser grade	0	56% (52–60)	64% (54–74)	NS	56% (48–64)	NS	48% (45–51)					69% (65–73)	45% (32–59)	0.0007	45% (32–59)	0.0007
	1	22% (19–25)	20% (12–28)		31% (23–39)		43% (40–46)					31% (27–35)	55% (41–68)		55% (41–68)	
	2	21% (18–24)	13% (6–20)		10% (5–15)		29% (26–32)									
Treatment intensity	0 Observation	1% (0–1)	100% (100–100)		0% (0–0)		1% (0–2)	100% (100–100)		0% (0–0)		6% (4–8)	100% (100–100)		0% (0–0)	
	1 PSSE (SEAS School)	14% (11–16)					41% (38–45)					60% (56–64)	0% (0–0)		100% (100–100)	
	2 Elastic brace (SpineCor)	9% (7–11)					10% (8–12)	0% (0–0)		100% (100–100)		8% (6–10)				
	3 Rigid brace 18/21 h/d	29% (26–33)	0% (0–0)		100% (100–100)		31% (28–34)					20% (17–24)				
	4 Rigid brace 22/24 h/d	11% (9–13)					5% (3–6)					2% (1–3)				
	5 Very rigid brace (Sforzesco) 18–21 h/d	9% (7–11)					7% (5–9)					3% (2–4)				
	6 Very rigid brace (Sforzesco) 22–24 h/d	28% (24–31)					5% (3–6)					1% (0–2)				

**Table 5:** Adherence of the entire population to the expected EBPA option.

Risser		0	1	2
Cobb Degrees	Intensity of Treatment			
10–20°	0	10%	6%	6%
	1	78%	80%	82%
	2	5%	4%	1%
	3	5%	10%	10%
	4	1%	0%	0%
	5	1%	1%	2%
	6	0%	0%	0%
21–30°	0	1%	1%	1%
	1	16%	18%	24%
	2	14%	9%	6%
	3	39%	45%	47%
	4	13%	7%	6%
	5	8%	11%	10%
	6	9%	8%	7%
31–40°	0	0%	0%	0%
	1	1%	0%	1%
	2	0%	1%	1%
	3	9%	7%	4%
	4	16%	15%	9%
	5	4%	17%	18%
	6	70%	59%	67%
Severe (41–45°)	0	0%	0%	0%
	1	0%	0%	0%
	2	0%	0%	0%
	3	0%	0%	0%
	4	2%	0%	0%
	5	0%	0%	7%
	6	98%	100%	93%

The treatment applied is listed for each clinical condition (defined according to the degree of scoliosis and Risser test—see Appendix A). The intensity of applied treatment follows this ordinal scale: 0. Observation, 1. PSSE (SEAS School), 2. Elastic brace (SpineCor), 3. Rigid brace 18/21 h/d, 4. Rigid brace 22/24 h/d, 5. Very rigid brace (Sforzesco) 18–21 h/d, 6. Very rigid brace (Sforzesco) 22–24 h/d. The yellow cells correspond to the expected EBPA. There are patients with treatment above or below the expected EBPA range. The choices performed for each subgroup are reported in Table 4 to compare with the benchmarked studies.

**Table 6:** Comparison of evidence-based personalised approach (EBPA) with benchmarked Randomised Controlled Trials (RCTs): Plastic Brace (PB) and Elastic Brace (EB) subgroups with the BrAIST [8] and SpineCor [9] studies, respectively.

Analysis	Groups		Relative Risk (RR) of Success			Number Needed to Treat (NNT)	
	EBPA	RCT	RR	IC95	*p*	NNT	IC95
Comparison with BrAIST Study							
Efficacy	treated EBPA	controls BrAIST	2.0	1.7–2.5	chi^2^ = 307.4 *p* < 0.001	2.0	1.7–2.5
	treated EBPA	treated BrAIST	1.4	1.2–1.5	chi^2^ = 141.8 *p* < 0.001	3.8	2.9–5.3
Intent to Treat	treated EBPA	controls BrAIST	1.8	1.5–2.3	chi^2^ = 97.5 *p* < 0.001	2.5	2.0–3.3
	treated EBPA	treated BrAIST	1.2	1.1–1.4	chi^2^ = 24.46 *p* < 0.001	6.3	4.2–12.5
Comparison with SpineCor Study							
Efficacy	treated EBPA	controls SpineCor	1.7	1.2–2.5	chi^2^ = 184.2 *p* < 0.001	2.4	1.6–4.8
	treated EBPA	treated SpineCor	2.9	1.7–4.9	chi^2^ = 377.0 *p* < 0.001	1.6	1.2–2.2
Intent to Treat	treated EBPA	controls SpineCor	3.5	2.0–6.1	chi^2^ = 103.1 *p* < 0.001	1.6	1.3–2.1
	treated EBPA	treated SpineCor	1.2	0.97–1.5	chi^2^ = 5.96 *p* = 0.05 NS	6.7	3.2–100

RR: Relative Risk; NNT: Number Needed to Treat; IC95: Interval of Confidence 95%. In EBPA, we considered the subgroups comparable to the populations of RCTs. We used the Relative Risk (RR) of success since all data in RCTs and EBPA were collected prospectively. A higher RR shows the probability for a patient to achieve better results with one treatment vs. the other. It was in this way also possible to compare our subgroups to the control groups of RCTs, showing the superiority of EBPA on natural history. Note that the relative risk of success is different for the two comparisons: in BrAIST RCT, it was defined as remaining below 50° [8], while in SpineCor one, it was remaining below 45° [9].

**Figure A1:**
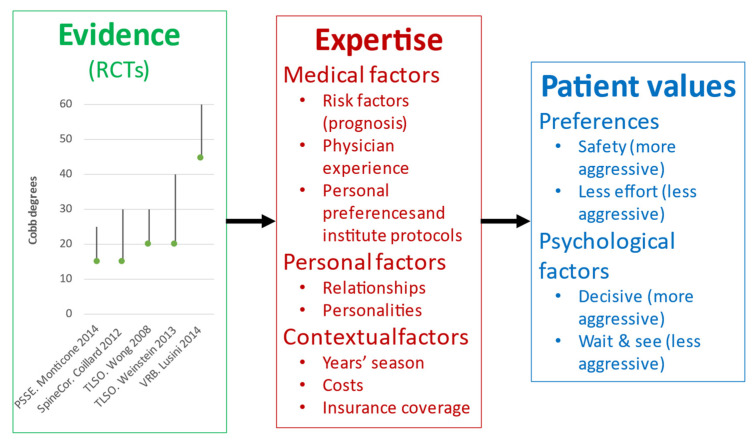
Graphical description of the three stages of an evidence-based approach according to the current clinical guidelines and the triad of evidence-based medicine used in the study. All details and explanations are reported in the Appendix A.

**Figure A2:**
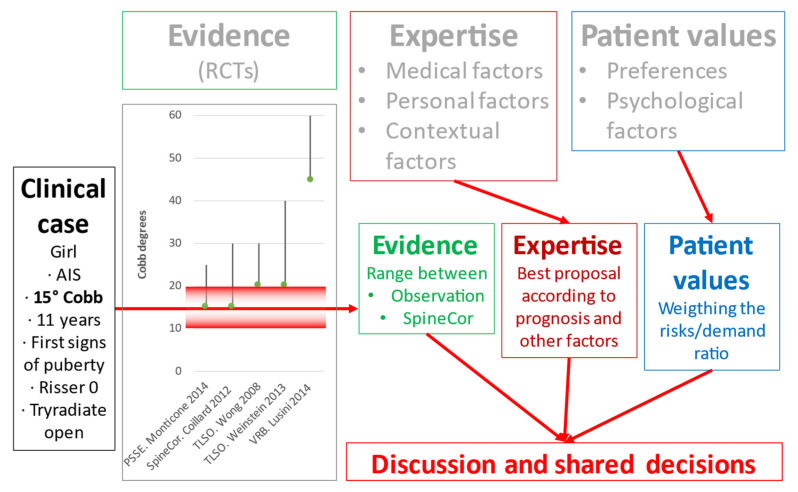
Application of an Evidence Based Clinical Approach to the theoretical Clinical case 1: age 11, Risser score 0, triradiate cartilage open, scoliosis of 15° Cobb.

**Figure A3:**
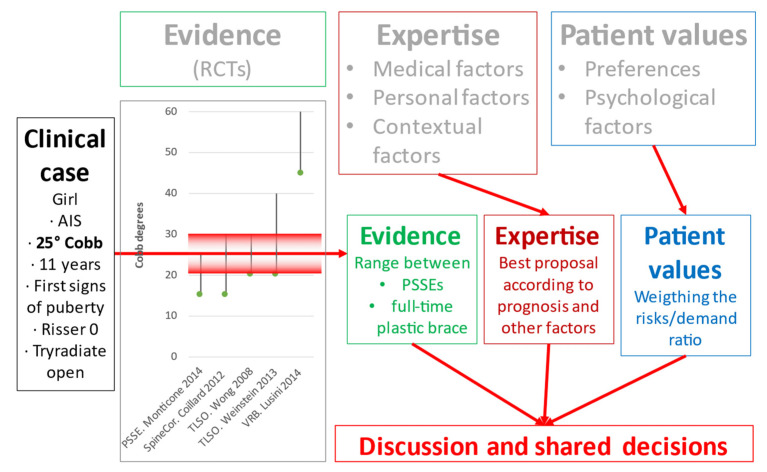
Application of an Evidence Based Clinical Approach to the theoretical Clinical case 2: age 11, Risser score 0, triradiate cartilage open, scoliosis of 25° Cobb.

**Figure A4:**
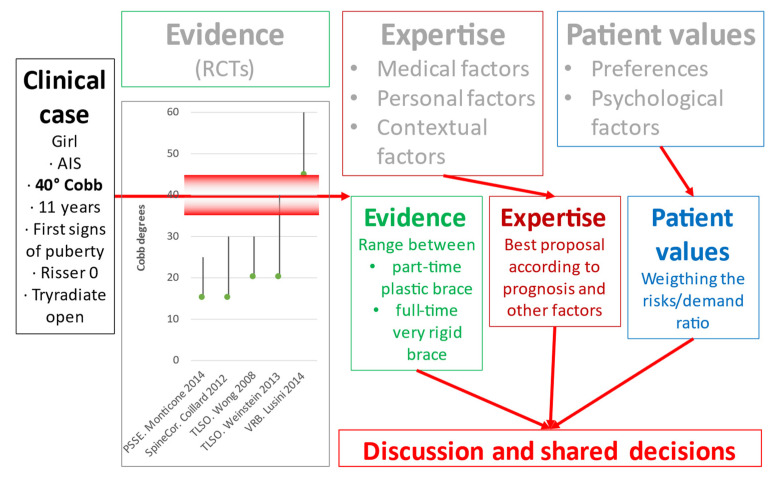
Application of an Evidence Based Clinical Approach to the theoretical Clinical case 3: age 11, Risser score 0, triradiate cartilage open, scoliosis of 40° Cobb.

## Data Availability

The data presented in this study are openly available in Zenodo at https://doi.org/10.5281/zenodo.5517156 (accessed on 20 September 2021).

## Appendix A. Description of the Evidence-Based Personalised Approach (EBPA) Used in This Study

Current Clinical Guidelines developed by the Society on Scoliosis Orthopedic and Rehabilitation Treatment (SOSORT) have been developed following an evidence-based clinical approach; they also followed a step-by-step approach to current treatments, where for each clinical condition, a different range of possible treatment could be proposed (Table A1) [11].

**Table A1:** Possible range of treatments proposed by the SOSORT Guidelines [11].

		Low (up to 20°)		Moderate (21–40°)		Severe (>40°)	
		Min	Max	Min	Max	Min	Max
Infantile		Obs 3	Obs 3	Obs 3	TTRB	TTRB	Su
Juvenile			PPSE	PSSE	FTRB	HTRB	
Adolescent	Risser 0	Obs 6	SSB	HTRB		TTRB	
	Risser 1			PSSE		FTRB	
	Risser 2						
	Risser 3						
	Risser 4	Obs12	SIR				
Adult up to 25 y		Nothing	PSSE	Obs12	SIR	Obs6	
Adult	No Pain			PSSE		Obs12	HTRB
	Pain	PSSE	SSB		HTRB	PSSE	Su
Elderly	No Pain	Nothing	PSSE	Obs36	PSSE	Obs12	HTRB
	Pain	PSSE	SSB	PSSE	HTRB	PSSE	Su
	Trunk decompensation	Obs6			PTRB		

For each clinical condition, treatment is proposed from a minimum (Min) to a maximum (Max) intensity. In progressive order of intensity, treatments are Obs: observation (with number of months from a minimum intensity of follow-up every 12 months to a maximum every 3 months); PSSE: Physiotherapeutic Scoliosis-Specific Exercises; SIR: Scoliosis Inpatient Rehabilitation; SSB: scoliosis soft brace; PTRB: part-time (12–15 h per day) rigid bracing; HTRB: half-time (16–18 h per day) rigid bracing; FTRB: full-time (19–22 h per day) rigid bracing; TTRB: total-time (23–24 h per day) rigid bracing; Su: surgery.

In this paper, we followed what was proposed by the SOSORT Guidelines, and we called our treatment an Evidence-Based Personalised Approach (EBPA). Consequently, per each clinical condition, we proposed a range of possible treatments according to Table A2 that is within the range proposed by SOSORT. Each physician in our Institute followed this protocol, and compliance was checked through our clinical data recording system. Regular monthly team meetings were held to improve the system and check variances–obviously accepted in individual cases.

**Table A2:** Range of treatments proposed in this study (EBPA) (yellow lines) compared to SOSORT Clinical Guidelines (white lines).

Guidelines	EBPA		Risser 0	Risser 1	Risser 2
Low (10–20°)		Minimum	Observation (6 months)		
	10–20°	Minimum	0. Observation (3 months)	0. Observation (6 months)	
		Maximum	2. Soft Scoliosis Brace		1. Physiotherapeutic Scoliosis Specific Exercises
		Maximum	Soft Scoliosis Brace		
Moderate (21–40°)		Minimum	Half-Time Rigid Bracing	Physiotherapeutic Scoliosis Specific Exercises	
	21–30°	Minimum	1. Physiotherapeutic Scoliosis-Specific Exercises		
		Maximum	5. Half-Time Very Rigid Bracing		
	31–40°	Minimum	3. Half-Time Rigid Bracing		
		Maximum	6. Full-Time Very Rigid Bracing		
		Maximum	Surgery		
Severe (>40°)		Minimum	6. Total-Time Rigid Bracing	6. Full-Time Rigid Bracing	
	41–45°	Minimum	6. Full-Time Very Rigid Bracing		
		Maximum	6. Total-Time Very Rigid Bracing		
		Maximum	Surgery		

The Moderate (21–40°) range of scoliosis of the Guidelines is split into two EBPA subgroups (21–30° and 31–40°). For each clinical condition, treatment is proposed by Guidelines and EBPA from a minimum (Min) to a maximum (Max) intensity. The SOSORT Guidelines propose the following intensity progression reported in this table: observation, Physiotherapeutic Scoliosis-Specific Exercises, Soft Scoliosis Brace, half-time (16–18 h per day) rigid bracing, full-time (19–22 h per day) rigid bracing, total-time (23–24 h per day) rigid bracing. In this study, the intensity follows the same progression but with a slight difference: 0 Observation, 1 PSSE (SEAS School), 2 Elastic brace (SpineCor), 3 Rigid brace 18/21 h/d, 4 Rigid brace 22/24 h/d, 5 Very rigid brace (Sforzesco) 18–21 h/d, 6 Very rigid brace (Sforzesco) 22–24 h/d.

Figure A1 reports a graphical description of the three stages of an evidence-based approach according to the current clinical guidelines and the triad of evidence-based medicine [11].

The first step of the EBPA is the evaluation of the clinical situation according to the current evidence. At this moment in time, for adolescents with idiopathic scoliosis (AIS), Risser score between 0 and 2, we have the RCTs reported in the figure and described in the main text: Monticone 2014 showing the efficacy of Physiotherapeutic Scoliosis-Specific Exercises (PSSE) above “wait & see” for curves 15–25° Cobb; Coillard 2012 showing the efficacy of SpineCor above “wait & see” for curves 15–30° Cobb; Wong 2018 showing the efficacy of plastic braces above SpineCor for curves 20–30°; Weinstein 2014 showing the efficacy of plastic braces above “wait & see” for curves 20–40° Cobb; Lusini 2012 showing the efficacy of very rigid plastic braces above “wait & see” for curves 45–60° Cobb with a reduction of the need of surgery of 50%.

The second step of the EBPA is to apply the clinical expertise of the treating clinicians. This takes into account multiple factors, including:

- Medical factors: they include commonly described risk factors determining the prognosis, even if without a complete certainty; physician experience drives to a more or less conservative or aggressive attitude; physicians’ preferences and institute protocols greatly influence the dosage (number of hours per day of treatment), since there are no strong scientific data on the topic.
- Personal factors: they include the relationships with patient and family, the evaluation of the personalities of patient and family, and how they could influence treatment.

Contextual factors: they include the years’ season (e.g., in the summer, plastic braces are more demanding), treatment costs, and insurance coverage.

The third step of EBPA is patients’ values. They include multiple factors, including the following:

- Preferences: the need for safety with as few risks as possible drives to a more aggressive attitude (more demanding treatments), while more significant attention to care and psychological well-being can lead to the opposite.
- Psychological factors: a more significant internal locus of control or an aggressive attitude will lead to more demanding treatments, while the opposite will become true on the other extreme of the psychological spectrum.

The physician will first evaluate the clinical situation and identify a range of possible treatments according to the current evidence and guidelines. Expertise will allow the clinician to “weigh” this range of possibilities according to the factors mentioned above. As a result, he/she will have a preferential proposal for the patient while providing all information to her/him and the family about the whole range of possibilities and the risk/demand ratio of each choice (a more demanding treatment decreases the risk of progression, and vice versa). According to the current knowledge, the pathology and progression probability are also described, including the current and future health risks. Finally, the clinician presents and discusses the prognosis and other “expertise-based” factors with their current strength of evidence. This process allows the patient and family to orientate the final decision, expressing their values in an interactive process, including open questions and answers sessions. Sometimes, the third step continues after the consultation, during the cognitive–behavioural session described in the text, and/or through other contacts in the subsequent days with clinicians. In addition, there could be an agreement for a time-buying strategy, i.e., a less demanding treatment with reduced follow-up time to closely check the outcomes and eventually change therapy as soon as possible.

In some cases, the situation is crystal clear, such as when there is no pathology (no need of treatment), or, at the other side of the spectrum, when there is a surgical case with the patient and family willing to try to avoid it (immediate most demanding treatment for the longest time). In these cases, there is no discussion; it is a black or white situation. On the contrary, in most cases, there are multiple possible options in terms of treatment or dosage. In these gray situations, there is plenty of space for discussion and shared decisions. The usual length of the first consultation, including this process and a proper history taking and clinical exam, lasts on average 30/40 min for an expert clinician, with a range between 50% and 200% of this time.

In Figure A2, Figure A3 and Figure A4, we exemplify the process described above. All three cases consider AIS of patients at age 11, Risser score 0, and triradiate cartilage open (i.e., maximum progression risk according to the current knowledge).

In case the scoliosis is of 15° Cobb (Figure A2), we have first to take into account the measurement error, which leads to a range between 10° and 20°. With these possibilities, we could consider a range of possible treatments from observation to SpineCor. Factors such as prominence, rigidity, sagittal plane, family history, and other prognostic elements will drive the clinician’s preference and proposal. The decision will finally be achieved by discussing it with the patient and family.

In case the scoliosis is of 25° Cobb (Figure A3), the measurement error leads to a range between 20° and 30°; the possible treatments range between PSSEs and full-time plastic rigid brace. The last could be proposed for example in case of an important prominence or rigidity, while the first in case of an important postural component with no big signs of deformity and perhaps being in full summer for a patient living at the seaside: in this case, a closer follow-up would also be proposed. The final decision will always rely on the patient and family risk/demand ratio evaluation.

Finally, in case of scoliosis at the top range of this study, with 40° Cobb (Figure A4) and a range between 35° and 45° to be considered, a brace is unavoidable according to the current evidence. The expertise will play a role in proposing a part-time (18 h per day) rigid plastic brace in case of good prognosis versus a full-time (23/24 h per day) very rigid brace in case of a patient considered at high risk of surgery due to other features (important prominence, high rigidity, big decompensation, flat back, etc). Contrarily to all the other cases, for the latter, very bad prognosis situation, very little space will be given to patient and family discussion.
